# Supplementary material for: A hidden confounder for microbiome studies: medications used years before sample collection
Source: mSystems. 2025 Sep 5;10(10):e00541-25. doi: 10.1128/msystems.00541-25 (PMC12542737; doi:10.1128/msystems.00541-25)
Supplement: Supplemental Figures — Figures S1 to S4. [file msystems.00541-25-s0001.docx]

Extended Data Figures
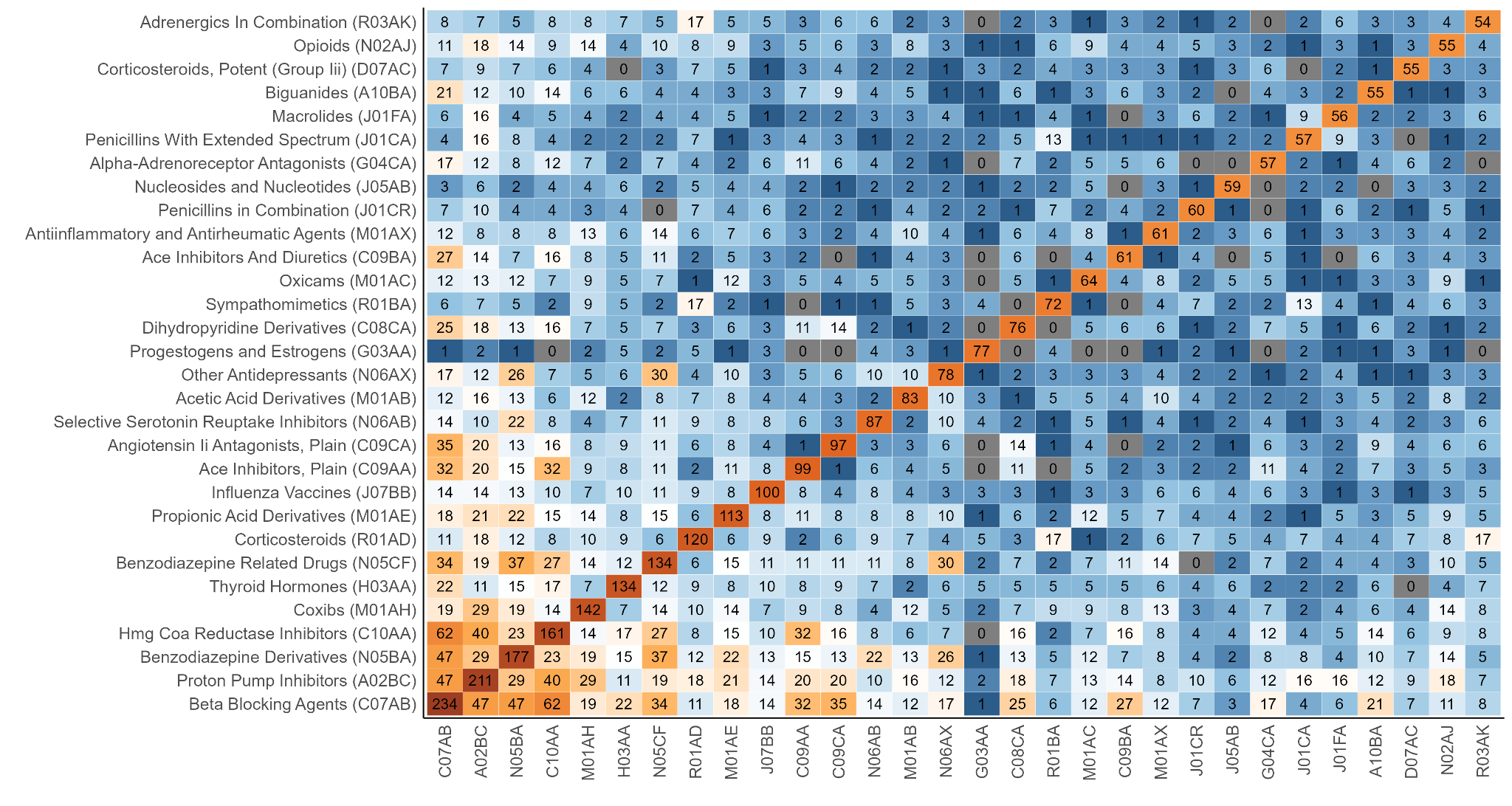


**Extended Data Fig. 1**. Number of participants using the drugs (ATC4-level) and drug combinations at T1. The number of participants using a specific drug is shown diagonally, and the number of subjects using the combination of the drugs is shown off-diagonally.

**
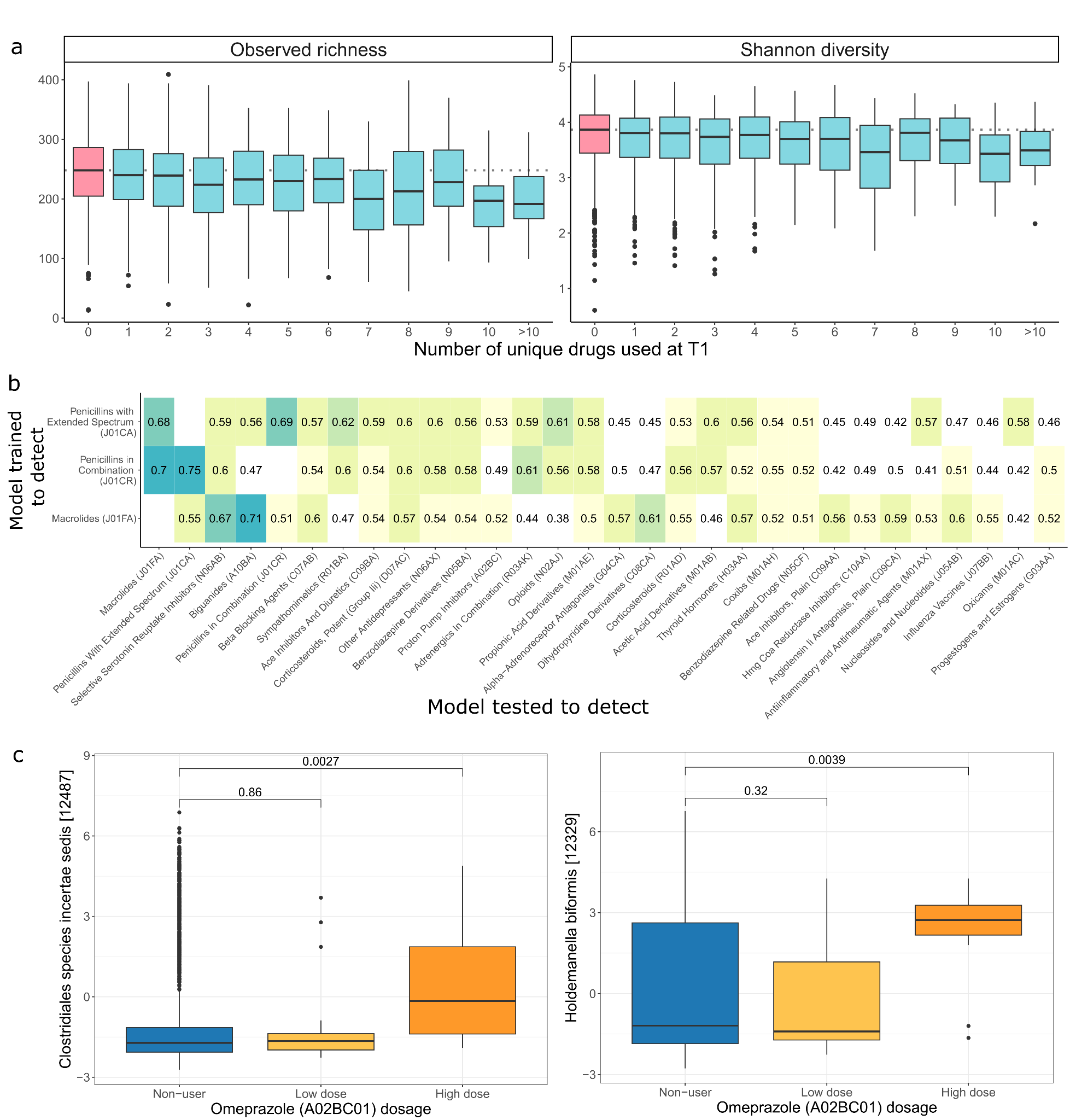
**

**Extended Data Fig. 2.** Active drug usage. (a) Alpha diversity measures (Observed richness and Shannon diversity) associated with the number of prescriptions at T1. (b) The performance of machine learning models aimed at detecting antibiotic usage is applied to detect the usage of the drugs. Values show the model's performance (Area Under the Receiver Operating Characteristics AUROC) in an independent test set AUROC. (c) Associations between selected individual species CLR-transformed abundance and PPI omeprazole dosage. Post-hoc t-test p-values are shown for the group comparisons.

**
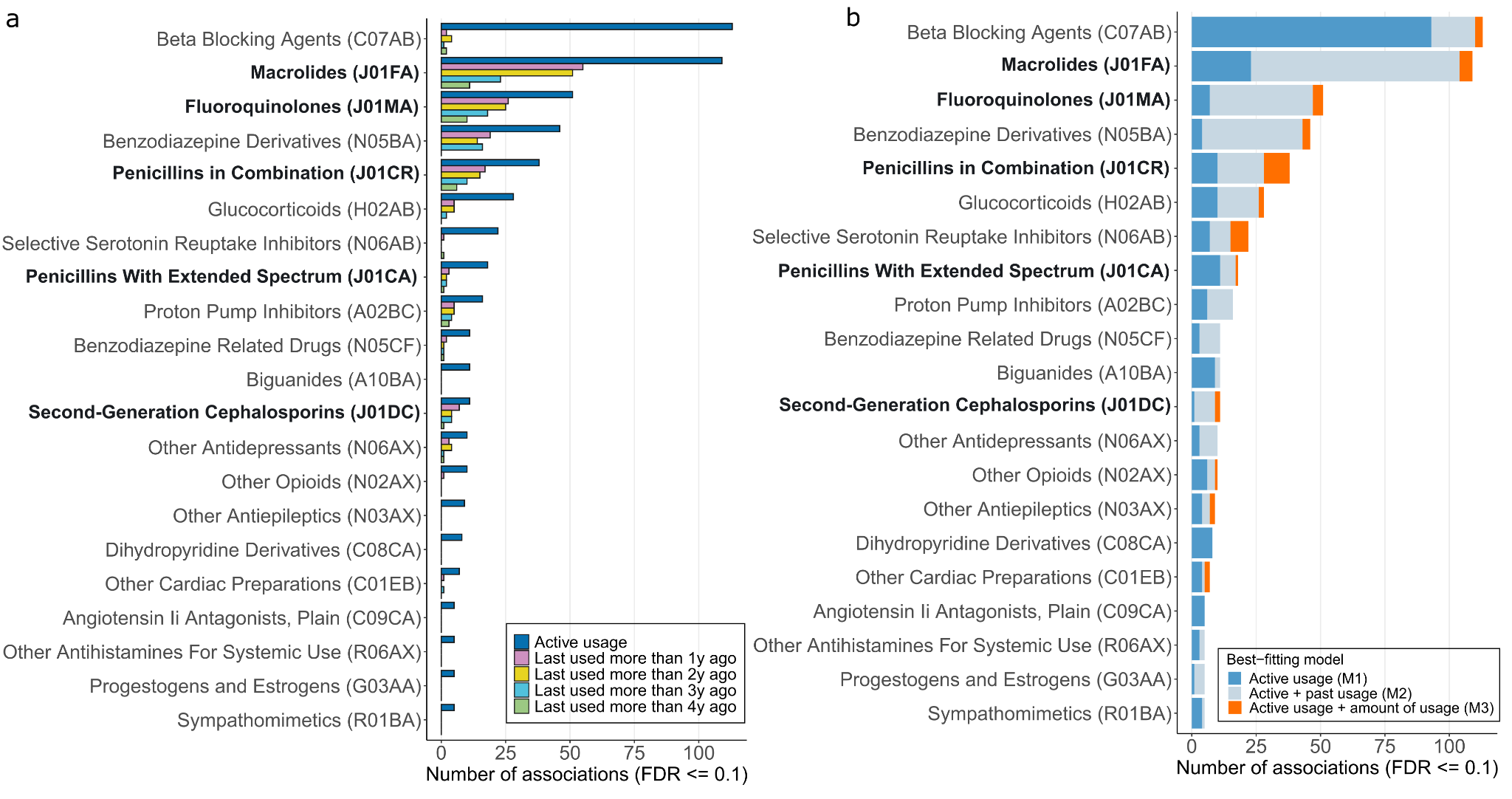
**

**Extended Data Fig. 3. N**umber of univariate associations with microbiome presence-absence (PA). (a) Drug carryover effects. The number of univariate associations between subjects not taking the drug and subjects having taken the drug more than 1 to 4 years prior to the microbiome sample collection. (b) Additive drug effects. The proportion of the univariate associations identified with active drug usage according to the model that best describes the association. In addition to active drug usage as a binary trait, active drug usage together with past drug usage as a binary trait or the amount of drug usage as a continuous trait are considered.

**
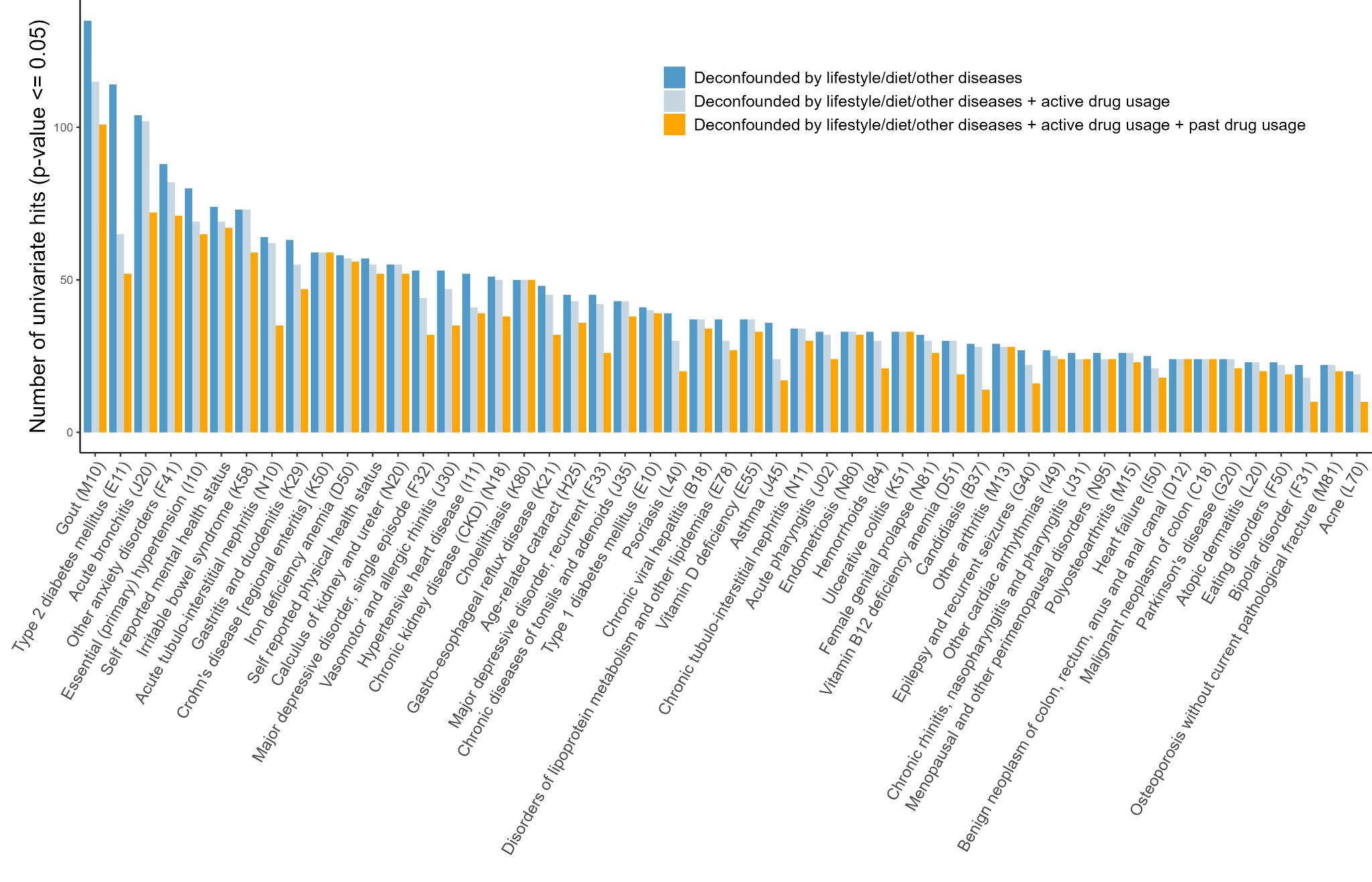
**

**Extended Data Fig 4.** Number of univariate associations between the CLR-transformed abundance of bacterial species and prevalent diseases after deconfounding analysis.
